# Spatiotemporal heterogeneity of the association between socioeconomic development and health policy attention: a geographically and temporally weighted regression modeling study in China

**DOI:** 10.3389/fpubh.2024.1338142

**Published:** 2024-08-27

**Authors:** Rongxin He, Hongchuan Wang, Wannian Liang

**Affiliations:** ^1^Vanke School of Public Health, Tsinghua University, Beijing, China; ^2^Institute for Healthy China, Tsinghua University, Beijing, China; ^3^School of Public Policy & Management, Tsinghua University, Beijing, China

**Keywords:** health policy attention, socioeconomic development, geographically and temporally weighted regression, spatiotemporal heterogeneity, COVID-19

## Abstract

**Objectives:**

Health policy attention (HPA) refers to the extent of attention given by governments to health issues in public policy and is generally influenced by socioeconomic development. This study aimed to examine the spatiotemporal heterogeneity and clustering of the associations between socioeconomic factors and HPA.

**Study design:**

Longitudinal study.

**Methods:**

This study examined the spatiotemporal heterogeneity of the association between public and provincial government attention, economic development, and demographic transition and HPA by using geographically and temporally weighted regression (GTWR). Word2Vec machine learning technology was utilized to calculate HPA data in 323 cities and independent variable data was collected in each city in China over the period of 2018–2021.

**Results:**

The results showed that there is a substantial overall rise in HPA levels throughout China following the COVID-19 pandemic. Furthermore, the GTWR results revealed significant spatiotemporal heterogeneity in the associations between HPA and public and provincial government attention, economic development, and demographic transition, particularly in the context of COVID-19. The impact of provincial government attention on HPA decreased from the capital of the political center outward, while the impact of public financial investment decreased in less developed cities during the pandemic. It was only cities with high levels of aging are more likely to receive greater HPA.

**Conclusion:**

The finding highlighted the remarkable spatial and temporal variations in the associations between the variables and HPA across different regions in China, emphasizing the need for region-specific policies to strengthen the focus on health by municipal governments.

## 1 Introduction

Health, defined by the World Health Organization (WHO), refers to all health-related topics, including diseases and conditions, health and wellbeing, health interventions, health behaviors, health systems, socio-political determinants, etc., (1). Health encompasses a wide range of topics, and health issues and their determinants often exhibit significant spatiotemporal heterogeneity. In recent years, numerous studies have adopted a spatiotemporal perspective to analyze health issues, thoroughly examining the spatiotemporal distribution characteristics of factors such as health resources and disease incidence (2–4). Based on these analyses, spatial econometric models have been used to establish the relationships between health outcomes and their influencing factors (5–7). Building on this foundation, this paper attempts to explore the spatiotemporal association between socioeconomic development and health policy attention.

The attention that governments pay to health issues in policymaking can have a profound impact on the health of populations (8, 9). Recent decades have witnessed increasing global attention to health affairs and the formulation of diverse health strategies, with the COVID-19 pandemic emphasizing the significance of health policy on a global scale (10–12). WHO has stressed the need for nations to prioritize population health protection in their policy agendas. This necessitates an examination of government responses to health issues and the link between Health Policy Attention (HPA) and influencing factors.

Past research indicates that government policy attention can be driven by factors such as economic development, population dynamics, and inter-government networks. For instance, the 2008 financial crisis led to a shift in government attention between healthcare and budget deficits (13). Regional population dynamics and inter-sectoral collaboration can also influence local government policy attention (14). Furthermore, public attention, institutional background, and party membership may impact policymakers’ policy focus (15–17). In the realm of health policy attention, limited exploration has been made into the factors influencing HPA. Some studies suggest that government attention to health topics can be responsive to public opinions, and the preferences of various parties, including organizations, policymakers, personal and mass media, can influence HPA (18–22). Additionally, directions from superior authorities and nearby cities influence the policy attention of subordinate governments on health (23).

Despite these findings, existing studies on HPA have not adequately explored the spatiotemporal heterogeneity of socioeconomic factors affecting city government policymaking, which is crucial when comparing the determinants of government HPA. While prior research has mainly focused on national-level data, HPA varies significantly at sub-national levels due to local context differences, implying that government HPA may be influenced by a variety of socioeconomic factors across different cities (10, 18, 19, 22, 24). Moreover, government policies exhibit varying trends at different stages, with the pandemic having a lasting impact on policy attention (10, 25, 26). Therefore, a comprehensive exploration of government HPA requires a robust spatiotemporal analysis.

This study used the definition of health that is promoted by the WHO, which is a broad conceptualization of health comprising many health-related topics (1). It also aimed to explore the spatiotemporal differentiation of the association between the government’s HPA and socioeconomic factors through GTWR analysis. It is particularly important in exploring the impact of socioeconomic development on the HPA in different cities or regions before and after the COVID-19 pandemic. However, there has been limited research identifying their association from a spatiotemporal perspective. This study aims to fill that gap.

## 2 Methods

### 2.1 Data

The HPA data of 323 Chinese cities and 31 provinces from 2018 to 2021 was obtained from the annual Government Work Report (GWR). Focusing on the Chinese context, GWR is a legally binding document and a significant policy instrument that documents the Chinese government’s responsibilities and achievements. The report outlines targets and plans for the government’s work and serves as the annual action program for the local government. Therefore, in this study, the use of literal expressions of health affairs in the GWR is employed as a proxy for the evaluation of HPA, in order to gain insight into the level of government attention dedicated to health policy in China. The text data for this study was sourced from the WinGo Financial text data platform. This study also employed the data from the China City Statistical Yearbook (2019–2022) and the National Economic and Social Development Statistical Bulletin (2019–2022) of each city, including population size and structure, economic development, air quality, city type and other socioeconomic characteristics of cities, reflect the social and economic development of cities, and other variables. The public attention data were acquired from the Baidu search index webside using the keyword “health” in Chinese for sample cities.

### 2.2 Variables

#### 2.2.1 Health policy attention

In this study, health policy attention refers to the government’s attention to health topics in formulating and implementing public policies. The indicator of HPA at the prefecture level from 2018 to 2021 is considered the dependent variable in assessing the level of attention that each city’s government pays to public health (Table 1). Organizational attention is often reflected in the changes in the frequency of the use of words (27). The proportion of health words in the total number of words in the GWR was used to measure the HPA. This approach is preferred as it can effectively avoid the influence of document size on the evaluation (28). Specifically, the construction process of the HPA indicator was as follows. First, a seed word set related to health in Chinese was created by combining the text of “Healthy China 2030 Strategic Plan”’ and drawing on the idea of building text indicators (29). Second, to address the problem of people using multiple words with similar semantics to describe the same concept, it was necessary to expand the seed word set of similar terms. The Word2Vec machine learning technology has recently been a landmark achievement in the field (30, 31). The core of this technology is the neural network Word Embedding method, which consists of representing words as multi-dimensional vectors based on contextual semantic information, thus allowing for the calculation of semantic similarity between words by computing the similarity between the vectors (32). For the purposes of this study, the Continuous Bag-of-words Model (CBOW Model) in Word2Vec was used to train the Chinese government work report corpus. CBOW model:


$$\max\underset{w\in C}{\sum }\log p\left(w|\mathrm{Context}\left(w\right)\right)$$

**Table 1:** Variable summary.

Variable		Mean value	Max value	Min value	Standard deviation
Dependent variable					
HPA	Health policy attention (%)	1.3089	2.5450	0.3906	0.2690
Independent Variables					
Attention from the public and provincial government					
PA	Public attention	0.9092	4.9465	0.0110	0.7022
PGA	Provincial government attention (%)	1.3136	2.0010	0.7740	0.2499
Demographic transition					
AGE65	The proportion of the population aged 65 or older (%)	13.3916	23.5260	2.9340	3.4073
POP	Population (million people)	4.2903	32.1243	0.2038	3.6394
UR	Urbanisation rate (%)	59.6278	99.7500	21.8700	13.7270
Economic development					
GDPPER	GDP *per capita* (ten thousand yuan)	6.3309	21.8118	1.2447	3.4921
BEPER	Budget expenditure of public finance *per capita* (ten thousand yuan)	1.3172	2.5450	0.3906	0.6893
Control Variables					
CT	City type (1 = high administrative level city, 0 = general city)	0.8869	1	0	0.3169
AQI	Air quality index	66.7891	126.6078	23.1096	18.9123

Where C represents corpus, w is the central word, and Context (w) indicates the context of the seed word. The Contextual Bag-of-Words (CBOW) model is a probability-based approach to predicting the current word according to its context. By maximizing the objective function, the word vector corresponding to the central word can be obtained, and then the similarity of the seed word can be calculated through vector similarity. This model has been trained on massive text corpora and has been proven to be an effective means of avoiding the subjectivity of artificial thesaurus definitions and the weak correlations of generic synonym tools. In order to determine the index word set, 411 words were selected after expert verification. To calculate the local government HPA index, the proportion of health-related term frequency in the total term frequency was multiplied by 100. The higher the value, the more critical the local government is to health.

#### 2.2.2 Attention from the public and provincial government

Public opinion plays a vital role in influencing public policy (33–38) and affects policymaking attention to health through short-term error correction and coexists with it in a long-term equilibrium (19). Meanwhile, public involvement initiatives or activities can increase decision-maker awareness in the healthcare sector and thus influence strategies and priority settings related to healthcare (39, 40). Furthermore, public attention manifested by social media and news can also influence policy attention in a more general, relational, and instantaneous way (41, 42). Online public opinions provide meaningful information for policymakers’ focus on public policy (43–45). Thus, the public attention on health topics could be a vital influencing factor on HPA.

Moreover, the policy attention of the provincial government may also influence the policy attention of the inferior government. The top-down vertical diffusion of policy attention has been observed between the federal and state governments (23, 46). The political influence was also found between Chinese central and local governments, while the latter retain a certain degree of autonomy (47–49). For instance, it is found that the attention allocation on safety management of different levels manifests a stable equilibrium in the long term (50). The urban land marketization policy from the central government was associated with local land marketization (51). Thus, the attention that higher-level governments give to health policy could increase the focus of local governments on the same issues. In this study, attention from the public and provincial government are considered the independent variables.

#### 2.2.3 Economic development

Economic development may influence the government’s HPA within the region under the government’s jurisdiction. Health service provision is unevenly distributed spatially between developed and developing countries (52, 53). In China, there are significant disparities in public health services owing to the substantially differentiated health expenditures across regions (54). Even though the central government dedicated fiscal inputs in health fields to the poor and western regions, the inequalities in the allocation of health resources are still widening in these areas (55). Also, indicators of local economic development, such as gross domestic product (GDP) *per capita*, are positively related to the health expenditure of local governments in China (56). Thus, it is hypothesized that HPA would be higher in cities with higher levels of economic development. In this study, GDP *per capita* and budget expenditure of public finance *per capita* at the prefecture level is selected as the main indicators of economic development in the spatiotemporal regression modeling.

#### 2.2.4 Demographic transition

Demographic transitions can potentially influence the government’s HPA. The aging of population has been dramatically accelerated in recent decades, and this is drawing significant attention from the government worldwide (57–59). Importantly, population aging is closely related to the government’s health expenditure (60). Moreover, the mass migration from rural to urban areas also raises health problems that need to be addressed. For example, the rapid urbanization process in China has attributed to a higher rate of poor health reporting (61). In addition, the population decline in many countries poses heavy burdens to fiscal budgets and, thus, healthcare provisions (62–64). This is important to contemporary China since its population declined in 2022 for the first time in the last 60 years (65). In this study, the proportion of the population aged 65 or older, permanent population size, and urbanization rate at the prefecture-level are considered the main indicators of demographic transition.

### 2.3 Spatiotemporal regression modeling

Geographically and temporally weighted regression (GTWR) offers a valuable alternative for exploring spatiotemporal heterogeneity that traditional regression models fail to capture. An increasing number of studies have demonstrated the efficacy of GTWR in housing (66, 67), air pollution (68, 69), and transportation (70). These studies have consistently found that the GTWR model produced a better model fit than naive/a-spatial models, suggesting its potential for informing health policy decisions amid the current pandemic. However, due to the highly computationally intensive nature of the GTWR model, its application is constrained to small size datasets, restricting its wider use. Nonetheless, the potential of GTWR for exploring spatiotemporal heterogeneity cannot be overlooked in the current context of dynamically changing HPA. It is thus critical to explore ways of improving the computational efficiency of the GTWR model and enabling its wider application in the social sciences. A typical GTWR model can be written as follows:


$$Y_{i}=\beta_{0}\left(u_{i},v_{i},t_{i}\right)+\underset{k}{\sum }\beta_{k}\left(u_{i},v_{i},t_{i}\right)X_{ik}+\varepsilon_{i}$$

Where Yi is the dependent variable percentage of health-related keywords in a government work report of the city i; (ui, vi, ti) denotes the spatial location (ui, vi as coordinates) of census tract i at time ti; β0 (ui, vi, ti) is the intercept value; βk (ui, vi, ti) represents a vector of parameter value for the independent variable k at the census tract I, and Xik is the respective independent variable; and εi denotes an error term for census tract i. What is distinct about the GTWR model is that it allows the parameters βk (ui, vi, ti) to vary across the model to measure both the spatial and temporal variations in a spatiotemporal dataset. To calibrate this model, a spacetime weight matrix W (ui, vi, ti), a diagonal matrix with elements representing the spatial and temporal weights of each census tract i, is required. The optimal spatiotemporal weight matrix can be determined through a cross-validation (CV) approach, which seeks to obtain the best goodness of fit. This process was executed using the local weighted least squares approach in combination with the GTWR model. Specifically, data preparation was conducted using the R programming language, whilst the model was calibrated with the ArcGIS GTWR add-in (66, 67).

Moran’s I was computed for the dataset, and its value of 0.163 (*p* < 0.001) demonstrated strong spatial autocorrelation, necessitating a spatial regression approach. A test for spatial nonstationarity was also conducted by comparing the interquartile from the GTWR with twice the standard errors from the ordinary least squares (OLS) model. The results indicated that all the variables exhibited extra local variations, making GTWR more suitable to explore the spatiotemporal heterogeneity (66). Furthermore, the GTWR model was also found to have a higher adjusted *R*-squared of 0.20 and a lower AICc of 105.576, compared to the OLS model values of 0.09 and 164.817, respectively. This suggested that the GTWR model significantly improved the overall model performance in reflecting the spatial and temporal variations in the research sample.

## 3 Results

### 3.1 Spatial characteristics of HPA across the study period

**Figure 1:**
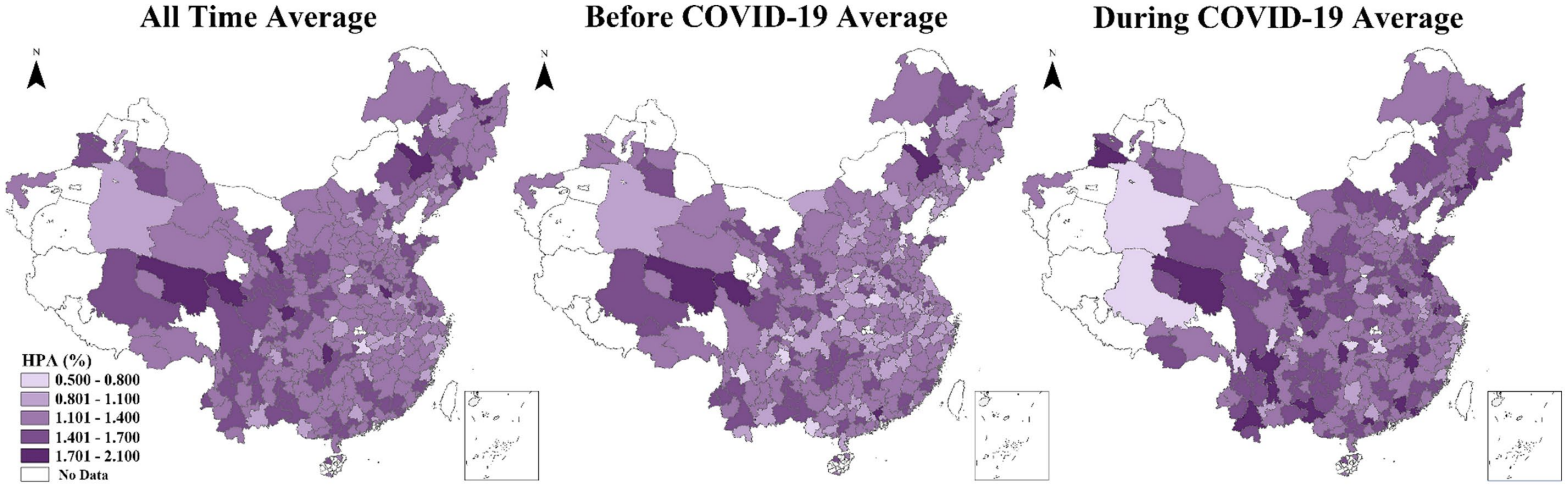
Spatial pattern of HPA in all time, before COVID-19, and during COVID-19 periods.

Spatial differences in HPA across the study period were observed in China (Figure 1), increasing from the east to the west with an average value of 1.308%. In general, regions with relatively low HPA (less than 1.1%) were mostly located in the eastern and middle parts of China, while regions with relatively high HPA (more than 1.4%) were mainly concentrated in the Qinghai-Tibetan Plateau area, the Northeast Plain area, and Yunnan-Sichuan provinces. The eastern and middle regions were also found to have relatively high HPA in Guangdong, Fujian, Shannxi, Henan, Shandong, and Jiangsu provinces. Further, the average HPA before COVID-19 was 1.221%, and this increased to 1.366% during the pandemic. Cities with relatively high HPA (more than 1.4%) noticeably increased in the study period, suggesting a substantial overall rise in HPA levels throughout China following the pandemic.

### 3.2 Geographically and temporally weighted regression modeling

#### 3.2.1 Overall model

The results of the GTWR model, presented in Table 2, suggest that public attention and provincial government attention had a positive impact on HPA. This finding is consistent with expectations, as increases in these variables are expected to increase HPA. Furthermore, the coefficient of the population was positive, indicating that increases in population size can lead to increases in HPA. However, the coefficient of proportion of the population aged 65 or older was negative, suggesting that increases in this variable can lead to decreases in HPA. The GTWR model results also revealed that urbanization rate, GDP *per capita*, and budget expenditure of public finance *per capita* all had different effects on HPA. Urbanization rate and GDP *per capita* had a negative impact, whereas budget expenditure of public finance *per capita* had a positive effect. Additionally, coefficients of city type and air quality index were both negative, suggesting that increases in these variables can lead to decreases in HPA.

**Table 2:** GTWR model summary.

Variable	AVG	MIN	LQ	MED	UQ	MAX
PA	0.0005	−0.0985	−0.0473	−0.0100	0.0376	1.3536
PGA	0.2064	−0.4581	0.1706	0.2318	0.2643	0.3250
AGE65	−0.0033	−0.0188	−0.0052	−0.0032	−0.0018	0.0228
POP	0.0014	−0.1891	−0.0038	0.0029	0.0054	0.0215
UR	−0.0006	−0.0034	−0.0015	−0.0008	−0.0003	0.0119
GDPPER	−0.0096	−0.0710	−0.0125	−0.0092	−0.0066	0.0082
BEPER	0.0187	−0.0859	0.0093	0.0194	0.0322	0.1652
CT	−0.0203	−0.5809	−0.0788	−0.0132	0.0323	0.8702
AQI	−0.0018	−0.0037	−0.0025	−0.0019	−0.0012	0.0014
AICc	105.576					
Adjusted R^2^	0.2015					

#### 3.2.2 Temporal dimension

Table 3 presents a comparison of the average coefficients of selected variables on HPA between the pre-COVID-19 and COVID-19 periods. Results show that the coefficients of public attention, provincial government attention, and budget expenditure *per capita* increased during the pandemic, indicating that the positive influence of these variables on HPA became stronger. At the national level, the coefficient of proportion of the population aged 65 or older and GDP *per capita* remained negative, requires further spatial analysis at sub-national level. On the other hand, the coefficients of population, city type, and air quality index exhibited varying changes during the pandemic.

**Table 3:** Average coefficients in different periods.

Variable	Before COVID-19 (2018–2019)	During COVID-19 (2020–2021)	Average coefficient change
PA	0.0002	0.0008	0.0006
PGA	0.1896	0.2169	0.0273
AGE65	−0.0035	−0.0031	0.0004
POP	0.0038	−0.0009	−0.0047
UR	−0.0011	−0.0001	0.0010
GDPPER	−0.0081	−0.0110	−0.0029
BEPER	0.0144	0.0226	0.0082
CT	0.0232	−0.0613	−0.0845
AQI	−0.0025	−0.0011	0.0014

#### 3.2.3 Spatial dimension

**Figure 2:**
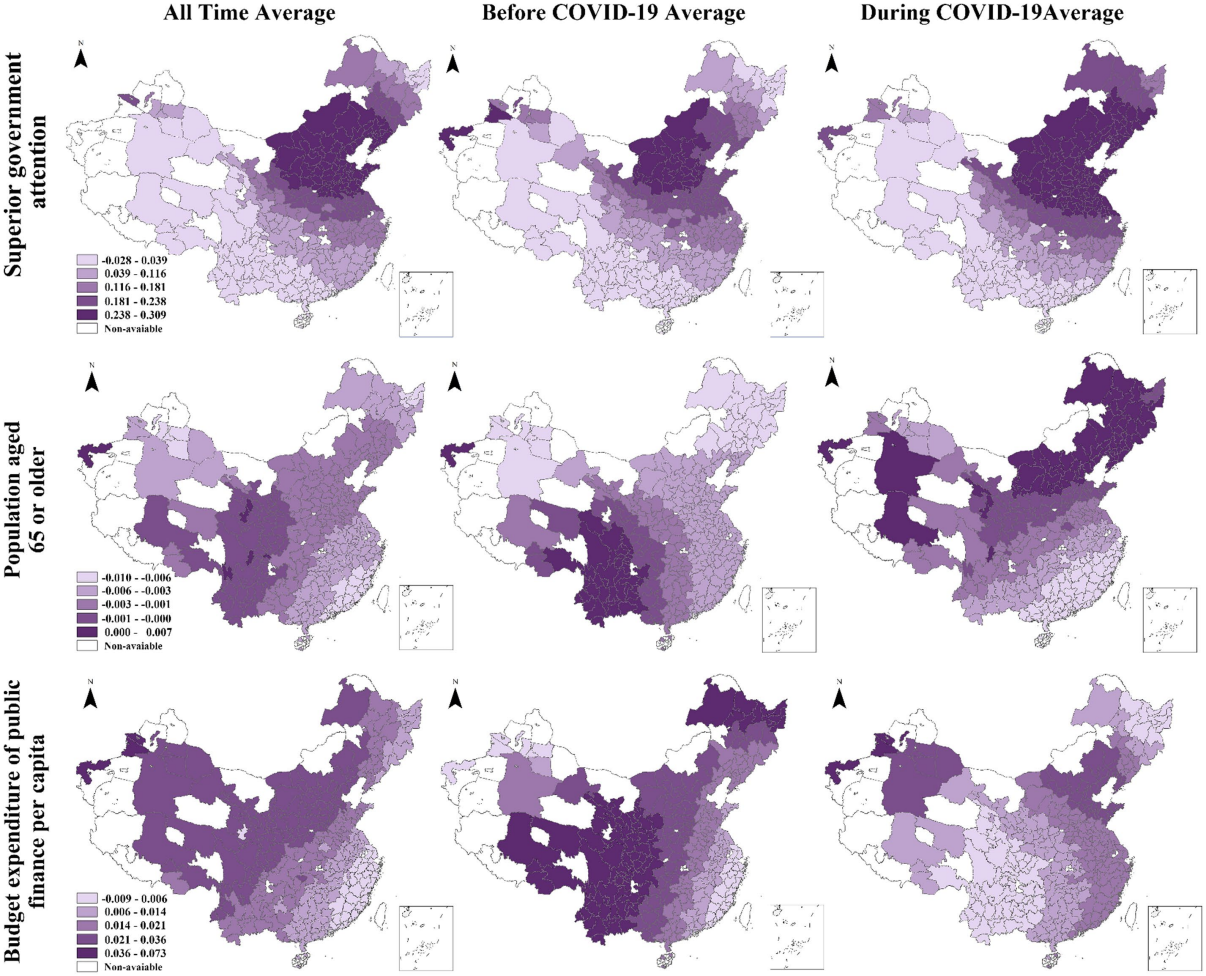
Spatial pattern of the average coefficients for three variables in all time, before COVID-19, and during COVID-19 periods.

Figure 2 indicates the spatial distribution characteristics of the association between socioeconomic factors and HPA. The average effects of provincial government attention vary geographically, with the highest coefficients observed in the Beijing-centered area, and diminishing coefficient levels observed in the surrounding regions throughout the study period. In contrast, the western regions display the weakest association with provincial government attention, with negative coefficients observed in some instances. Prior to the COVID-19 pandemic, the strongest impact of provincial government attention was concentrated in Beijing and its neighboring provinces, such as Hebei and Nei Monggol. However, during the pandemic, this effect expanded to other provinces, including Liaoning, Shanxi, and Shandong, ultimately resulting in a broader geographic impact. The average effects of aging on various outcomes exhibits a distinct spatial pattern. Specifically, the aging effect is consistently negative across all time periods, with the highest coefficients observed in the western regions, gradually declining toward the east. Prior to the outbreak of COVID-19, the southwestern regions demonstrated the greatest vulnerability to aging, whereas after the pandemic, the northeastern regions experienced the most significant aging effect. This effect ultimately weakened moving toward the southwest region. The average effects of budget expenditure and public finance *per capita* during the study period are strongest in the northwestern regions and diminish gradually southeastward. Before COVID-19, the coefficients of public budget expenditure were the largest in the western area and some northeastern regions. And the coefficients decrease eastward and become negative on the southeast coast. During COVID-19, on the contrary, the effects of public budget expenditure are most significant in eastern areas and then diminish westward.

## 4 Discussion

This study aimed to investigate the spatiotemporal heterogeneity in the association between HPA and socioeconomic development in China, where marked differences in HPA have been observed across the country. The results revealed that public and provincial government concern, economic development, and demographic transition were significantly associated with HPA. These findings suggest that the spatial heterogeneity of the association between HPA and socioeconomic factors should be taken into account when developing health policies. Moreover, it may be necessary to develop different policies or investments to increase HPA in different regions. Overall, the findings of this study had several important ramifications for health policy and future research.

First, these findings suggest that provincial government health policy attention has a strong influence on local government decision-making. Notably, the positive association between government health concerns and city-level health policy attention has been further strengthened during the COVID-19 pandemic, with an increased effect of provincial government attention on cities’ HPA. This might be due to the climbing public attention during the pandemic and the top-down public health emergency response (71–73). This study also found that the most robust average coefficients for provincial government attention were in the Beijing-centered areas. This may be due to Beijing is political center in China, as it has the most potent political influence and receives the most resources from the central government (50). During the COVID-19 pandemic, political influence and access to resources expanded to a broader range of areas, indicating that increased pressure from the provincial government in health policy may encourage local governments to pay more attention to health issues. These findings suggest that public health crises have played an important role in strengthening intergovernmental relations, and this impact is more pronounced in cities and regions closer to the political center.

Second, this study examines the association between demographic transition and HPA in cities. Contrary to this study’s initial hypothesis, the results reveal a negative association between urbanization and HPA, suggesting that high levels of urbanization may lead to decreased policy attention to health due to the growing pressure on local governments to address various issues (74). This study also found interesting results regarding the association between aging populations and HPA. It was only in cities with high levels of aging (southwest and northeast China), saw a greater increase in HPA in response to its aging population. Notably, the study found a close-to-zero negative coefficient for this variable in the all-time period, indicating that the positive and negative coefficients of all cities in China even out. These findings provide valuable insights into the significant difference between demographic transition and HPA in cities, suggest that cities with a high proportion of older adult residents are more likely to receive greater attention in health policy, but the extent of this attention may vary depending on the region.

Third, the study shows a significant correlation between economic development, as measured by GDP *per capita*, and public finance budget expenditure *per capita*, with HPA. Unexpectedly, the results indicate that higher GDP *per capita* is negatively associated with HPA. This may be due to cities with high GDP *per capita*, the government may direct its attention toward more priorities, including unemployment, public security, ecological environment, etc. (75, 76). However, budget expenditure of public finance *per capita* is positively correlated with HPA. This association has been strengthened during the COVID-19 pandemic due to increased government investment in public health. Results from this study also highlight a significant difference between the less-developed western region and the highly developed eastern region, suggesting that the effects of economic factors on HPA may vary across cities, especially during the COVID-19 pandemic. Before the pandemic, less developed cities in western region relied more heavily on public finance expenditures to improve their healthcare systems, while more developed cities may pay less attention to health in their public finance. However, with the outbreak of COVID-19, cities became more reliant on public finance to address public health emergencies. The economic resourcefulness of the more developed cities provided them with greater potential to raise their policy attention to health during the pandemic (77, 78). When less developed cities or regions face public health crises, strengthening HPA requires a higher level of public financial investment than before.

This study has several limitations that should be acknowledged. Firstly, the data on the socioeconomic variables were limited to average statistical values, and only a limited number of socioeconomic variables were examined. Detailed information on the distribution of economic development within each city was not available. As a result, the R-squared value for this study is moderately high. Future studies should aim to consider a wider range of factors, such as the economic structure, natural environment, and official characteristics. This would help to provide a more comprehensive understanding of the association between socioeconomic variables and health outcomes. By incorporating additional variables and methods of analysis, it may be possible to strengthen the explanatory power of the model and increase the understanding of this complex relationship.

## 5 Conclusion

This study utilized a GTWR model to explore the heterogeneity and clustering of socioeconomic factors associated with HPA in cities in China, with a focus on the impacts of the ongoing COVID-19 pandemic. The results showed that there is a substantial overall rise in HPA levels throughout China following the COVID-19 pandemic. The pandemic has played a significant role in strengthening intergovernmental relations, with the impact of provincial government attention being more pronounced on HPA in cities closer to the political center. The economic resourcefulness provided developed cities with greater potential to increase their HPA during the pandemic. In contrast, less developed cities require a higher level of public financial investment to strengthen HPA. It was only in cities with high levels of aging are more likely to receive greater HPA. The findings highlighted the remarkable spatial and temporal variations in the associations between the variables and HPA across different regions in China, emphasizing the need for region-specific policies to strengthen the focus on health by municipal governments. These findings offer valuable insights into the heterogeneity and clustering of determinants related to HPA, which can inform effective policy-making in response to the pandemic.

## Data Availability

The original contributions presented in the study are included in the article/Supplementary materials, further inquiries can be directed to the corresponding author/s.

## References

[1] WHO. (2022). WHO launches first ever global report on infection prevention and control. Available at: https://www.who.int/news/item/06-05-2022-who-launches-first-ever-global-report-on-infection-prevention-and-control (Accessed March 24, 2023)

[2] Chen H Zhao LY Yu J. Spatiotemporal evolution of healthcare service capacity at township health centers in China. Front Public Health. (2023) 11:1229453. doi: 10.3389/fpubh.2023.1229453, PMID: 38145066 PMC10739490

[3] Daw MA El-Bouzedi AH Ahmed MO. The epidemiological and spatiotemporal characteristics of the 2019 novel coronavirus disease (COVID-19) in Libya. Front Public Health. (2021) 9:628211. doi: 10.3389/fpubh.2021.628211, PMID: 34195168 PMC8236517

[4] El Deeb O. Spatial autocorrelation and the dynamics of the mean center of COVID-19 infections in Lebanon. Front Appl Math Statistics. (2021) 6:620064. doi: 10.3389/fams.2020.620064

[5] Bonzani C Scull P Yamamoto D. A spatiotemporal analysis of the social determinants of health for COVID-19. Geospat Health. (2023) 18:1153. doi: 10.4081/gh.2023.1153 37246546

[6] Qiu LJ Yang LS Li HR Wang L. The impact of health resource enhancement and its spatiotemporal relationship with population health. Front Public Health. (2023) 10:1043184. doi: 10.3389/fpubh.2022.1043184 36699901 PMC9868711

[7] She YY Chen QY Ye S Wang P Wu BB Zhang SY. Spatial-temporal heterogeneity and driving factors of PM2.5 in China: a natural and socioeconomic perspective. Front Public Health. (2022) 10:1051116. doi: 10.3389/fpubh.2022.1051116 36466497 PMC9713317

[8] Bao R Liu TL. How does government attention matter in air pollution control? Evidence from government annual reports. Resources Conserv Recycl. (2022) 185:106435. doi: 10.1016/j.resconrec.2022.106435

[9] Zhang W Zhou Y Li J Zeng T Liao J. Does the attention of the Chinese government influence Chinese nutrition, exercise, and health? Based on the content analysis of the central government work reports from 1978 to 2020. Front Nutr. (2021) 8:724176. doi: 10.3389/fnut.2021.724176, PMID: 34733874 PMC8558527

[10] Cheng Q Kang J Lin M. Understanding the evolution of government attention in response to COVID-19 in China: a topic modeling approach. Healthcare. (2021) 9:898. doi: 10.3390/healthcare9070898 34356277 PMC8304999

[11] Jansen D Kosola S Arevalo LC Gaspar de Matos M Boode K Saxena S. Child and adolescent health needs attention now, and in the aftermath of the COVID-19 pandemic. Int J Public Health. (2020) 65:723–5. doi: 10.1007/s00038-020-01446-8, PMID: 32740686 PMC7395573

[12] Nepomnyashchiy L Dahn B Saykpah R Raghavan M. COVID-19: Africa needs unprecedented attention to strengthen community health systems. Lancet. (2020) 396:150–2. doi: 10.1016/S0140-6736(20)31532-4, PMID: 32682465 PMC7365630

[13] Harper S. Economic and social implications of aging societies. Science. (2014) 346:587–91. doi: 10.1126/science.1254405 25359967

[14] Qi X Han S. The way toward sustainability: policy attention evolution of Chinese local governments to promote entrepreneurship of returnees based on grounded theory and social network analysis. Sustain For. (2022) 14:13283. doi: 10.3390/su142013283

[15] Fan S Xue L Xu J. What drives policy attention to climate change in China? An empirical analysis through the lens of people’s daily. Sustain For. (2018) 10:2977. doi: 10.3390/su10092977

[16] Hemphill L Russell A Schöpke-Gonzalez AM. What drives US congressional members’ policy attention on twitter? Policy Internet. (2021) 13:233–56. doi: 10.1002/poi3.245

[17] Peterson HL. Narrative policy images: intersecting narrative & attention in presidential stories about the environment. Policy Stud J. (2021). doi: 10.1111/psj.12447

[18] Albalawi Y Sixsmith J. Agenda setting for health promotion: exploring an adapted model for the social media era. JMIR Public Health Surveill. (2015) 1:e5014. doi: 10.2196/publichealth.5014 PMC4869225 27227139

[19] Jennings W John P. The dynamics of political attention: public opinion and the Queen's speech in the United Kingdom. Am J Polit Sci. (2009) 53:838–54. doi: 10.1111/j.1540-5907.2009.00404.x

[20] Kaltoft MK Turner R Cunich M Salkeld G Nielsen JB Dowie J. Addressing preference heterogeneity in public health policy by combining cluster analysis and multi-criteria decision analysis: proof of method. Heal Econ Rev. (2015) 5:1–11. doi: 10.1186/s13561-015-0048-4 PMC4429422 25992305

[21] Paolucci F Mentzakis E Defechereux T Niessen LW. Equity and efficiency preferences of health policy makers in China—a stated preference analysis. Health Policy Plan. (2015) 30:1059–66. doi: 10.1093/heapol/czu123, PMID: 25500745

[22] Sato H. Agenda setting for smoking control in Japan, 1945-1990: influence of the mass media on national health policy making. J Health Commun. (2003) 8:23–40. doi: 10.1080/10810730305731, PMID: 12635809

[23] Pacheco J Boushey G. Public health and agenda setting: determinants of state attention to tobacco and vaccines. J Health Polit Policy Law. (2014) 39:565–89. doi: 10.1215/03616878-2682612, PMID: 24603088

[24] Wu X Shi L Lu X Li X Ma L. Government dissemination of epidemic information as a policy instrument during COVID-19 pandemic: evidence from Chinese cities. Cities. (2022) 125:103658. doi: 10.1016/j.cities.2022.103658, PMID: 35264817 PMC8891011

[25] Auerbach JD Forsyth AD Davey C Hargreaves JR Group for lessons from pandemic HIV prevention for the COVID-19 response. Living with COVID-19 and preparing for future pandemics: revisiting lessons from the HIV pandemic. Lancet Hiv. (2023) 10:E62–8. doi: 10.1016/S2352-3018(22)00301-0, PMID: 36370713 PMC9764384

[26] Camilletti E Nesbitt-Ahmed Z. COVID-19 and a ‘crisis of care’: a feminist analysis of public policy responses to paid and unpaid care and domestic work. Int Labour Rev. (2022) 161:195–218. doi: 10.1111/ilr.12354 35602284 PMC9111651

[27] Weber RP. Basic content analysis. 2rd ed. London: Sage (1990).

[28] Loughran T Mcdonald B. When is a liability not a liability? Textual analysis, dictionaries, and 10‐Ks. J Financ. (2011) 66:35–65. doi: 10.1111/j.1540-6261.2010.01625.x

[29] Li F. Textual analysis of corporate disclosures: a survey of the literature. J Account Lit. (2010) 29:143–65.

[30] LeCun Y Bengio Y Hinton G. Deep learning. Nature. (2015) 521:436–44. doi: 10.1038/nature14539 26017442

[31] Mikolov T. Sutskever I. Chen K. Corrado G.S. Dean J. (2013). Distributed representations of words and phrases and their compositionality. Adv Neural Inf Proces Syst 2, 3111–3119.

[32] Bengio Y Ducharme R Vincent P Jauvin C. A Neural Probabilistic Language Model. J Mach Learn Res. (2003) 3:1137–55. doi: 10.1162/153244303322533223

[33] Hafner T Shiffman J. The emergence of global attention to health systems strengthening. Health Policy Plan. (2013) 28:41–50. doi: 10.1093/heapol/czs023 22407017

[34] Newig J. Public attention, political action: the example of environmental regulation. Ration Soc. (2004) 16:149–90. doi: 10.1177/1043463104043713

[35] Page BI Shapiro RY. Effects of public opinion on policy. Am Polit Sci Rev. (1983) 77:175–90. doi: 10.2307/1956018

[36] Soroka SN Wlezien C. Opinion–policy dynamics: public preferences and public expenditure in the United Kingdom. Br J Polit Sci. (2005) 35:665–89. doi: 10.1017/S0007123405000347

[37] Wright GC Erikson RS McIver JP. Public opinion and policy liberalism in the American states. Am J Polit Sci. (1987) 31:980. doi: 10.2307/2111232

[38] Wlezien C Soroka SN. Political institutions and the opinion–policy Link. West Eur Polit. (2012) 35:1407–32. doi: 10.1080/01402382.2012.713752

[39] Conklin A Morris ZS Nolte E. Involving the public in healthcare policy: An update of the research evidence and proposed evaluation framework. Santa Monica: RAND Corporation (2010).

[40] Mitton C Smith N Peacock S Evoy B Abelson J. Public participation in health care priority setting: a scoping review. Health Policy. (2009) 91:219–28. doi: 10.1016/j.healthpol.2009.01.005, PMID: 19261347

[41] Murphy J Link MW Childs JH Tesfaye CL Dean E Stern M. Social media in public opinion research: executive summary of the Aapor task force on emerging Technologies in Public Opinion Research. Public Opin Q. (2014) 78:788–94. doi: 10.1093/poq/nfu053

[42] McGregor SC. Social media as public opinion: how journalists use social media to represent public opinion. Journalism. (2019) 20:1070–86. doi: 10.1177/1464884919845458

[43] Bonsón E Torres L Royo S Flores F. Local e-government 2.0: social media and corporate transparency in municipalities. Gov Inf Q. (2012) 29:123–32. doi: 10.1016/j.giq.2011.10.001

[44] Ceron A Negri F. The “social side” of public policy: monitoring online public opinion and its mobilization during the policy cycle: monitoring online public opinion during the policy cycle. Policy Internet. (2016) 8:131–47. doi: 10.1002/poi3.117

[45] Lev-On A Steinfeld N. Local engagement online: municipal Facebook pages as hubs of interaction. Gov Inf Q. (2015) 32:299–307. doi: 10.1016/j.giq.2015.05.007

[46] Lowery D Gray V Baumgartner FR. Policy attention in state and nation: is anyone listening to the Laboratories of Democracy? Publius J Feder. (2011) 41:286–310. doi: 10.1093/publius/pjq039

[47] Gao J. Pernicious manipulation of performance measures in China's cadre evaluation system*. China Q. (2015) 223:618–37. doi: 10.1017/S0305741015000806

[48] Suisheng Z. China’s central-local relationship: a historical perspective. New York: Routledge (2019).

[49] Xu C. The fundamental institutions of China's reforms and development. J Econ Lit. (2011) 49:1076–151. doi: 10.1257/jel.49.4.1076

[50] Xu J Lu L Wei J. Hierarchical difference in attention allocation of local governments: explaining change and stability in safety management. Saf Sci. (2022) 152:105789. doi: 10.1016/j.ssci.2022.105789

[51] Liu T Cao G Yan Y Wang RY. Urban land marketization in China: central policy, local initiative, and market mechanism. Land Use Policy. (2016) 57:265–76. doi: 10.1016/j.landusepol.2016.06.001

[52] Costa-Font J Pons-Novell J. Public health expenditure and spatial interactions in a decentralized national health system. Health Econ. (2007) 16:291–306. doi: 10.1002/hec.1154, PMID: 16981194

[53] Signorelli C Odone A Bianco D Di Vivo N Bevere F. Health expenditure for prevention in Italy, 2006-2013: descriptive analysis, regional trends and international comparisons. Epidemiol Prevenzione. (2016) 40:374–80. doi: 10.19191/EP16.5.AD01.095 27764919

[54] Yan J Xu L Tan Y. The spatial pattern and dynamic evolution of public health expenditure in China. Econ Geogr. (2017) 37:82–91.

[55] Jin H Qian X. How the Chinese government has done with public health from the perspective of the evaluation and comparison about public-health expenditure. Int J Environ Res Public Health. (2020) 17:9272. doi: 10.3390/ijerph17249272, PMID: 33322428 PMC7764182

[56] Wang M Tao C. Research on the efficiency of local government health expenditure in China and its spatial spillover effect. Sustain For. (2019) 11:2469. doi: 10.3390/su11092469

[57] Bloom DE Chatterji S Kowal P Lloyd-Sherlock P Mckee M Rechel B. Macroeconomic implications of population ageing and selected policy responses. Lancet. (2015) 385:649–57. doi: 10.1016/S0140-6736(14)61464-1 25468167 PMC4469267

[58] Fang EF Scheibye-Knudsen M Jahn HJ Li J Ling L Guo H. A research agenda for aging in China in the 21st century. Ageing Res Rev. (2015) 24:197–205. doi: 10.1016/j.arr.2015.08.003, PMID: 26304837 PMC5179143

[59] Zhang NJ Guo M Zheng X. China: awakening giant developing solutions to population aging. Gerontologist. (2012) 52:589–96. doi: 10.1093/geront/gns105, PMID: 22936537

[60] De Meijer C Wouterse B Polder J Koopmanschap M. The effect of population aging on health expenditure growth: a critical review. Eur J Ageing. (2013) 10:353–61. doi: 10.1007/s10433-013-0280-x, PMID: 28804308 PMC5549212

[61] Van de Poel E O'Donnell O Van Doorslaer E. Is there a health penalty of China's rapid urbanization? Health Econ. (2012) 21:367–85. doi: 10.1002/hec.1717 21341344

[62] McCann. Urban futures, population ageing and demographic decline. Camb J Reg Econ Soc. (2017) 10:543–57. doi: 10.1093/cjres/rsx009

[63] Reher DS. Towards long-term population decline: a discussion of relevant issues. Eur J Popul. (2007) 23:189–207. doi: 10.1007/s10680-007-9120-z

[64] Shrestha LB. Population aging in developing countries: the elderly populations of developing countries are now growing more rapidly than those in industrialized nations, thanks to health advances and declining fertility rates. Health Aff. (2000) 19:204–12. doi: 10.1377/hlthaff.19.3.204 10812800

[65] Wang X. (2023). China's overall population falls in 2022. Available at: http://global.chinadaily.com.cn/a/202301/17/WS63c60d34a31057c47ebaa14d.html (Accessed February 1, 2023)

[66] Fotheringham AS Crespo R Yao J. Geographical and temporal weighted regression (GTWR). Geogr Anal. (2015) 47:431–52. doi: 10.1111/gean.12071

[67] Huang B Wu B Barry M. Geographically and temporally weighted regression for modeling spatio-temporal variation in house prices. Int J Geogr Inf Sci. (2010) 24:383–401. doi: 10.1080/13658810802672469

[68] Chu HJ Bilal M. PM 2.5 mapping using integrated geographically temporally weighted regression (GTWR) and random sample consensus (RANSAC) models. Environ Sci Pollut Res. (2019) 26:1902–10. doi: 10.1007/s11356-018-3763-7, PMID: 30460650

[69] Mirzaei M Amanollahi J Tzanis CG. Evaluation of linear, nonlinear, and hybrid models for predicting PM 2.5 based on a GTWR model and MODIS AOD data. Air Qual Atmos Health. (2019) 12:1215–24. doi: 10.1007/s11869-019-00739-z

[70] Ma X Zhang J Ding C Wang Y. A geographically and temporally weighted regression model to explore the spatiotemporal influence of built environment on transit ridership. Comput Environ Urban Syst. (2018) 70:113–24. doi: 10.1016/j.compenvurbsys.2018.03.001

[71] Gong X Han YY Hou MC Guo R. Online public attention during the early days of the COVID-19 pandemic: Infoveillance study based on Baidu index. JMIR Public Health Surveill. (2020) 6:225–37. doi: 10.2196/23098 PMC7584450 32960177

[72] Gao J Zhang P. Mechanisms of the Chinese Government's efforts to fight COVID-19: integration of top-down interventions and local governance. Health Security. (2022) 20:348–56. doi: 10.1089/hs.2021.0161, PMID: 35787156

[73] Liao QY Yuan JH Dong MH Yang L Fielding R Lam WWT. Public engagement and government responsiveness in the communications about COVID-19 during the early epidemic stage in China: Infodemiology study on social media data. J Med Internet Res. (2020) 22:e18796. doi: 10.2196/18796, PMID: 32412414 PMC7284407

[74] Zhao ZX Pan Y Zhu J Wu JX Zhu R. The impact of urbanization on the delivery of public service-related SDGs in China. Sustain Cities Soc. (2022) 80:103776. doi: 10.1016/j.scs.2022.103776

[75] Ardiyono SK Patunru AA. The impact of employment protection on FDI at different stages of economic development. World Econ. (2022) 45:3679–714. doi: 10.1111/twec.13299

[76] Shi T Yang SY Zhang W Zhou Q. Coupling coordination degree measurement and spatiotemporal heterogeneity between economic development and ecological environment ----empirical evidence from tropical and subtropical regions of China. J Clean Prod. (2020) 244:118739. doi: 10.1016/j.jclepro.2019.118739

[77] Colombage SRN Barua S Nanayakkara M Colombage UN. COVID-19 effects on public finance and SDG priorities in developing countries: comparative evidence from Bangladesh and Sri Lanka. Eur J Dev Res. (2023) 35:85–111. doi: 10.1057/s41287-022-00558-6, PMID: 35915624 PMC9330931

[78] Imam T Uddin S. How do economic and public finance statuses affect policy responses during a pandemic? –learning from the COVID-19 first wave. BMC Public Health. (2022) 22:1–11. doi: 10.1186/s12889-022-13209-6 35440081 PMC9016378

