# Supplementary material for: Spatiotemporal heterogeneity of the association between socioeconomic development and health policy attention: a geographically and temporally weighted regression modeling study in China
Source: Front Public Health. 2024 Aug 27;12:1338142. doi: 10.3389/fpubh.2024.1338142 (PMC11384576; doi:10.3389/fpubh.2024.1338142)
Supplement: Supplementary file 1 [file Table_1.DOCX]

**Table S1. Spatial Nonstationarity Tests of Variables**

| Variable | Interquartile (GTWR) | 2×SE (OLS) | Extra local variation |
| --- | --- | --- | --- |
| PA | 0.0849 | 0.058 | YES |
| PGA | 0.0937 | 0.042 | YES |
| AGE65 | 0.0034 | 0.004 | YES |
| POP | 0.0092 | 0.008 | YES |
| UR | 0.0012 | 0.002 | YES |
| GDPPER | 0.0059 | 0.039 | YES |
| BEPER | 0.0229 | 0.008 | YES |
| CT | 0.1111 | 0.070 | YES |
| AQI | 0.0013 | 0.000 | YES |

**Table S2. Summary of OLS Regression**

| Variable | Estimate | Std. Error |
| --- | --- | --- |
| Intercept | 1.175*** | 0.083 |
| PA | 0.005 | 0.029 |
| PGA | 0.243*** | 0.031 |
| AGE65 | -0.001 | 0.002 |
| POP | 0.000 | 0.004 |
| UR | 0.001 | 0.001 |
| GDPPER | -0.014*** | 0.004 |
| BEPER | 0.027** | 0.013 |
| CT | -0.02 | 0.017 |
| AQI | -0.002*** | 0.000 |
| Adjusted R2 | 0.088 | |

Note: *** p<0.001; ** p<0.01; * p<0.05; . p<0.1.
